# Relationship between childhood stressful events and drug consumption among university students

**DOI:** 10.1192/j.eurpsy.2024.522

**Published:** 2024-08-27

**Authors:** S. Rey-March, C. Giralt, S. Font-Mayolas, F. Calvo

**Affiliations:** ^1^Departament de Pedagogia, Institut de Recerca sobre Qualitat de Vida; ^2^Institut Català de la Salut, Centre d’Atenció Primària Blanes 2; ^3^Departament de Psicologia, Institut de Recerca sobre Qualitat de Vida, Girona, Spain

## Abstract

**Introduction:**

The purpose of this study is to investigate the potential relationship between stressful events experienced in childhood and subsequent toxic substance consumption among university students majoring in Social Education at a Spanish university during the academic year 2022-2023.

**Objectives:**

The primary objective is to analyze whether an association exists between stressful life events in childhood and patterns of substance consumption among university students.

**Methods:**

A cross-sectional, observational, and analytical design was employed. The target population encompassed 258 students enrolled in the Social Education program in 2023. The final sample consisted of 161 students. A questionnaire incorporating the Childhood Trauma Questionnaire - Short Form (CTQ-SF) and the Severity of Dependence Scale (SDS) was administered to assess trauma history and substance consumption.

**Results:**

A high percentage (95.03%) of students reported having consumed toxic substances at some point in their lives. The most common substances were alcohol (95.03%) and cannabis (52.8%). A statistically significant correlation was observed between childhood emotional abuse and increased alcohol consumption currently (p = 0.015). Furthermore, a significant relationship was identified between childhood sexual abuse and heightened alcohol consumption (p = 0.015). Moreover, positive correlations were found between sexual abuse and the consumption of specific drugs, such as cocaine and psychopharmaceuticals (p < 0.05).

No statistically significant differences were observed in drug consumption with regard to other forms of childhood maltreatment, such as emotional or physical neglect.

**Conclusions:**

The results underscore the connection between childhood stress experiences and substance consumption among university students. Emotional and sexual abuse in childhood are linked to higher alcohol consumption and, in some cases, specific drugs like cocaine and psychopharmaceuticals. These findings emphasize the importance of considering traumatic experiences when addressing prevention and treatment strategies for substance consumption among young student populations.

**Disclosure of Interest:**

None Declared

